# An International Marine-Atmospheric ^222^Rn Measurement Intercomparison in Bermuda Part I: NIST Calibration and Methodology for Standardized Sample Additions

**DOI:** 10.6028/jres.101.005

**Published:** 1996

**Authors:** R. Collé, M. P. Unterweger, P. A. Hodge, J. M. R. Hutchinson

**Affiliations:** National Institute of Standards and Technology, Gaithersburg, MD 20899-0001 USA

**Keywords:** calibration, environment, intercomparison, marine atmosphere, measurement, radium-226, radon-222, standards

## Abstract

As part of an international ^222^Rn measurement intercomparison conducted at Bermuda in October 1991, NIST provided standardized sample additions of known, but undisclosed (“blind”) ^222^Rn concentrations that could be related to U.S. national standards. The standardized sample additions were obtained with a calibrated ^226^Ra source and a specially-designed manifold used to obtain well-known dilution factors from simultaneous flow-rate measurements. The additions were introduced over sampling periods of several hours (typically 4 h) into a common streamline on a sampling tower used by the participating laboratories for their measurements. The standardized ^222^Rn activity concentrations for the intercomparison ranged from approximately 2.5 Bq · m^−3^ to 35 Bq · m^−3^ (of which the lower end of this range approached concentration levels for ambient Bermudian air) and had overall uncertainties, approximating a 3 standard deviation uncertainty interval, of about 6 % to 13 %. This paper describes the calibration and methodology for the standardized sample additions.

## 1. Introduction

In October 1991, an international measurement intercomparison of instruments used to measure trace atmospheric concentrations of ^222^Rn (radon) was organized by Drexel University and conducted at the Atmosphere/Ocean Chemistry Experiment (AEROCE) test site located at Tudor Hill, Bermuda which is operated in conjunction with the Bermuda Biological Station for Research, Inc. This intercomparison comprised two parts: *Viz*. (1) measurement comparisons among four laboratories of commonly sampled ambient air over approximately a 2 week test period; and, (2) measurement comparisons among three of these four laboratories of a select number of introduced samples with known radon activity concentration.

Measurements of atmospheric radon in remote marine environments are used to obtain information on the temporal and spatial distributions, which are in turn used to test and validate global models that simulate the transport and removal of trace atmospheric species from continental air masses. Unlike other chemical species, radon is an excellent tracer for such studies because it has a well-characterized source (large land masses from which radon diffuses ubiquitously) and only one principal “sink” (radioactive decay). For such measurement data to be useful, however, particularly when collected by different laboratories at different global sites using diverse instruments that are based on widely different collection and measurement principles, it is necessary that the collected data have at least a common relative, if not an absolute, reference basis. The intent of this intercomparison was to provide that basis for the participating laboratories.

The participating laboratories in the intercomparison were: Environmental Measurements Laboratory, U.S. Department of Energy (New York, USA); Drexel University (Philadelphia, USA); Australian Nuclear Science and Technology Organization (Menai NSW, Australia); and the Centre des Faibles Radioactivitis, Laboratoire Mixte C.N.R.S. – C.E.A. (Gif-sur-Yvette, France). Henceforth referred to as Lab E, Lab D, Lab A, and Lab F, respectively. The first three laboratories performed simultaneous measurements from a common streamline on an ambient air sampling tower. Lab F, in contradistinction, sampled ambient air nearly adjacent to the inlet of the sampling tower and therefore only participated in the ambient air intercomparisons.

The National Institute of Standards and Technology (NIST) was invited to participate in this intercomparison to serve as the “referee” and, if possible, to provide an “absolute” (sic) reference basis for the participants’ measurements. The planned scheme for the latter role consisted of NIST being able to provide standardized sample additions of known, but undisclosed (“blind”) radon concentrations that could be related to U.S. national standards. The standardized sample additions were to be introduced over sampling periods of several hours into a common streamline on the sampling tower used by the participants for their measurements. Beside being a “blind” intercomparison in terms of the radon activity concentrations, the timing and duration of the standard sample additions were also largely unknown to the participating laboratories. Radon concentrations for the sample additions were planned to vary from approximately a few times typical ambient concentration levels up to about 1000 times ambient. This necessitated being able to provide well-known sample dilutions over a wide dynamic range.

The sample additions were made with a commercially-available, solid ^226^Ra source, calibrated by NIST in terms of the available ^222^Rn concentration as a function of flow rate through the source. The source was employed in conjunction with a sampling and dilution gas-handling manifold also provided by NIST. “Grab samples” from the sampling streamline were also taken with sample bulbs that were returned to NIST for assaying the ^222^Rn concentration. These “grab samples” were taken as confirmatory measurements to insure that the source and manifold were not performing differently at the test site as at NIST where the source calibrations were made.

This paper summarizes and describes in detail the experimental arrangements, the NIST calibrations, methodology for the standardized additions, and the protocol for the experimental aspects of the intercomparison. The results of the intercomparison measurements for the participating laboratories are presented in a separate companion paper [1].

## 2. Instrumentation and Methodology

**Fig. 1:**
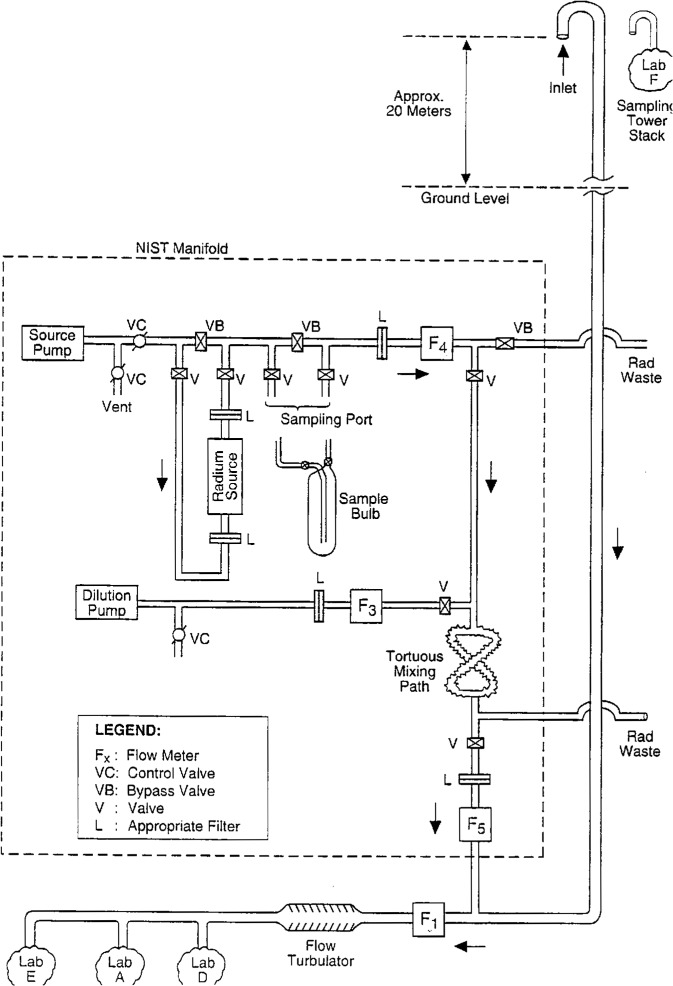
Schematic diagram of the experimental configuration used for the measurement intercomparison showing the NIST manifold that was employed to provide standardized additions of known radon concentration and the relative sampling locations for the participating laboratories.

The experimental arrangement used for the intercomparison is illustrated in the schematic diagram, Fig. 1.

Ambient air was sampled continuously from the top of a sampling tower located at the Tudor Hill, Bermuda test site. The sampling stack consisted of a nominal 10.2 cm diameter aluminum tube whose inlet was approximately 20 m above ground level. The inlet at the top of the tube was a goose-neck, which was inverted to minimize the intake of rain, and covered with a plastic mesh to prohibit the intake of birds, insects, etc. A similar diameter polyvinyl chloride (PVC) tube was coupled to the aluminum tube about 2.5 m above a horizontal section of the stack that was used by the participants. Air flow within the tube was maintained by sampling pumps that were part of the measurement instrumentation for three of the participating laboratories (Labs A, D, and E). These instruments were located at the terminus of the horizontal section. One laboratory (Lab F) performed measurements at the top of the sampling tower nearly adjacent to the inlet and, as mentioned previously, did not participate in the intercomparison of standardized additions. The standardized additions of known ^222^Rn activity concentration, and with known dilution factors, were made into the sampling stack at ground level at the foot of the sampling tower as shown in Fig. 1.

The NIST manifold was designed to obtain known radon concentrations from a calibrated ^226^Ra source, to provide standardized dilutions of this concentration, and to introduce them into the sampling streamline. The manifold also allowed the filling of sample bulbs that were used for confirmatory measurements of the provided radon concentration. The manifold was constructed, for the most part, of 1/4 in and 3/8 in (0.635 cm and 0.953 cm) stainless steel (SS) tubing with compression Swaglok^1^ and IPT threaded pipe fittings. All of the valves were of high-vacuum, stainless steel construction. The two pumps were simple, vibrating-diaphragm, aquarium pumps which had maximum flow rates of approximately 1.5 L· min^−1^ and which utilized ambient air outside their housings. The two control valves (VC in Fig. 1) located after the source pump were used to vary and control the flow rate through the radium source. With the use of appropriate triad of valves, the flow rate could be adjusted with the streamline bypassing the source. A similar triad of valves was used at the sampling port to either bypass the port or to direct the streamline of air through a sample bulb. The ^226^Ra source was a commercially-available, flow-through source which was independently calibrated by NIST and will be subsequently described in Sec. 3 of this paper. The flow rates through the radium source (*f*_4_) was measured with flow meter F_4_. The streamline after meter F_4_ could be either bypassed to a waste stream or directed to the sampling stack for a standard addition. The flow rate (*f*_3_) from the dilution pump was adjusted with a single, control valve teed off its streamline. This rate was measured with meter F_3_. Flows *f*_4_ and *f*_3_ were added and mixed through a tortuous mixing path that consisted of a crossed path of flexed SS bellows tubing which served as a flow turbulator. Its efficacy and the adequacy of mixing were demonstrated by confirmatory measurements that will be reported in Sec. 5 of this paper. A portion of the combined (*f*_4_ + *f*_3_) flow rate could be diverted through a control valve to a second waste line. The net flow rate (*f*_5_) after this diversion was measured with meter F_5_. The three meters (F_4_, F_3_, and F_5_) where matched and intercalibrated mass flow meters (Matheson Gas Products, East Rutherford, NJ) having a range of 0 L · min^−1^ to 3 L · min^−1^ with standard volume conditions of 21 °C and 760 mm Hg (1.013 × 10^5^ Pa), and having a reported calibration accuracy of 1.0 %. Confirmatory measurements of their *in situ* intercalibration are also reported in Sec. 5 of this paper. The two waste streamlines, which at times contained appreciable radon concentrations, were directed by flexible, plastic hose to distances considerably away from the sampling stack. For the first few standard additions, the waste hose outlet was located 30 m to 40 m southeast of the tower base, and was subsequently relocated about 30 m down the hill from the tower (which would place the outlet 40 m to 50 m below and west of the intake at the top of the tower). None of the diversions were considered to have appreciably altered any ambient radon concentration levels.

The outlet of the NIST manifold with flow rate *f*_5_ was directed into the main sampling stack where the flow of the combined streamlines was measured with meter F_1_ and mixed with a commercial, high-efficiency flow tubulator prior to sampling by the instrumentation of Labs A, D, and E. As specified by the manufacturer, meter F_1_ was a 0 ft^3^ · min^−1^ to 50 ft^3^ · min^−1^ (0 L · min^−1^ to 1416 L · min^−1^ mass flow meter (TSI, Inc., St. Paul, MN) with standard volume conditions of 70 °F (20.1 °C) and 14.7 lb · in^−2^ (1.013 × 10^5^ Pa) and reported “tolerance” of ±2 % of reading plus 0.2 % of full scale.

Throughout the intercomparison of standardized additions, all four flow meters were continuously monitored. Their 0 V to 5 V analog outputs were sampled at 10 s intervals and converted to digital signals with a 4096 channel analog-to-digital converter (ADC) and chronologically recorded on a dedicated personal computer (PC). This every 10 second file of all of the flow rates provided an exhaustively complete record of all flow-rate changes, and thereby the necessary dilution factors, throughout the course of all the standardized additions. The chronological record was maintained with the internal computer clock. This clock was cursorily intercompared with the timing devices of the measurement instruments of the participating laboratories. All clocks were in agreement within a minute or two, and any of the differences in clock times could be demonstrated to have negligible effect on the intercomparison results.

**Fig. 2:**
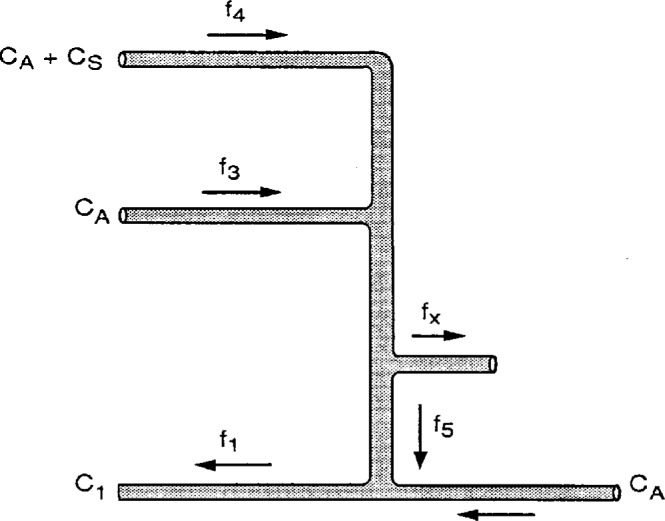
Simplified flow network for the experimental configuration shown in Fig. 1.

A simplified schema of the flow network is shown in Fig. 2. The radon concentration in the source and dilution lines are

$$C_{4}=C_{S}+C_{A}$$ (1)

and

$$C_{3}=C_{A},$$ (2)

respectively, where *C*_A_ is the ^222^Rn activity concentration in ambient air and *C*_S_ is the ^222^Rn activity concentration from the radium source when maintained at flow rate *f*_4_ (see Sec. 3). With *C*_4_ at flow rate *f*_4_ and *C*_3_ at flow rate *f*_3_, the concentration at their confluence is just the weighted sum:

$$\begin{array}{c} C=\frac{f_{4}C_{4}+f_{3}C_{3}}{\left(f_{4}+f_{3}\right)}\\ \;=\left[\frac{f_{4}}{f_{4}+f_{3}}\right]C_{S}+C_{A}. \end{array}$$ (3)

With a partial diversion of the combined flow rate (*f*_4_ + *f*_3_) to *f*_x_ and *f*_5_, the concentration exiting from the NIST manifold streamline is diluted to

$$\begin{array}{c} C_{1}=\left[\frac{f_{5}}{f_{1}}\right]C+\left[\frac{f_{1}-f_{5}}{f_{1}}\right]C_{A}\\ \;=\left[\frac{f_{5}}{f_{1}}\right]\;[\frac{f_{4}}{f_{4}+f_{3}}]C_{S}+C_{A} \end{array}$$ (4)

in the main sampling line. This is the radon concentration sampled by the participating laboratories. For discussion purposes, let us call the portion contributed by the standard sample addition *C*_0_, and call the combined flow-rate ratios the dilution factor *D*, so that:

$$C_{1}=C_{0}+C_{A};$$ (5)

$$C_{0}=D\;C_{S};$$ (6)

and

$$D=\left[\frac{f_{5}}{f_{1}}\right]\;\left[\frac{f_{4}}{f_{4}+f_{3}}\right].$$ (7)

It should be noted that there are two simplified situations: with *f_x_* = 0 and *f*_3_ = 0, *f*_4_ = *f*_5_ and the dilution factor is just *D* = (*f*_5_/*f*_1_) = (*f*_4_/*f*_1_). Alternatively, with *f_x_* = 0, (*f*_3_ + *f*_4_) = *f*_5_ and *D* = (*f*_4_/*f*_1_). For confirmatory purposes, both cases were tested in the series of standardized additions (see Sec. 5).

The protocol for initiating the standardized additions consisted of: (1) adjusting flow-rate *f*_4_ while bypassing the radium source; (2) opening the flow *f*_4_ through the source but diverting it out the waste line to allow time to reach a steady-state concentration *C*_S_ from the source and to flush the source line; (3) adjusting flow rates *f*_3_ and *f*_5_ with the appropriate control valves; and finally, (4) after all the flow rates are stabilized, and at the commencement of the standard addition, opening the valve after meter F_4_ and closing the waste bypass valve to direct *C*_S_ into the manifold stream. The standardized additions were typically of 4 h duration (with one exception) to allow sufficient overlap with the measurement intervals for each of the participating laboratories. Each addition was initiated at the start of one of the hourly measurement cycles of Lab E. The rapid and abrupt change in flow-rate *f*_1_ at those hourly intervals (a flow-rate change of approximately 400 L · min^−1^ over approximately 40 s as the Lab E pump went off and on again) provided a convenient internal timing mechanism. The standard addition was terminated, again at the end of one of the measurement cycles of Lab E, by: (1) rapidly diverting *C*_S_ from the main streamline to the bypass waste line; and (2) readjusting the flow rates (and thereby the dilution factor) for the next standard addition.

## 3. Calibration of the ^226^Ra Source

For a ^226^Ra source having activity *A*_Ra_, the instantaneous rate of production of ^222^Rn activity, *A*_Rn_, is simply given by

$$\frac{dA_{\text{Rn}}}{dt}=R=A_{\text{Ra}}\lambda_{\text{Rn}}$$ (8)

where *λ*_Rn_ = 2.09 × 10^−6^ s^−1^ is the decay constant for ^222^Rn. This result may be derived from several straightforward approaches, including taking the time-differential limit of the generalized Bateman equation steady-state solution for the growth of a radioactive daughter from decay of its parent.

Now, for an idealized flow-through ^226^Ra source, under continuous constant-flow conditions with completely mixed, turbulent air, the expected ^222^Rn activity concentration *C*_Rn_ in the source’s exiting streamline would be

$$C_{\text{Rn}}=R\frac{\phi }{f}=A_{\text{Ra}}\lambda_{\text{Rn}}\frac{\phi }{f}$$ (9)

where *ϕ* is the emanation fraction (i.e., the fraction of the total ^222^Rn generated in the source that is released into the air stream) and *f* is the flow rate through the source. This approach of obtaining the radon activity concentration in the streamline by dividing the continuously available radon activity by a corresponding flow rate is, of course, simplistic. Firstly, it ignores any time dependence necessary to reach a steady-state concentration *C*_Rn_ in the streamline. This, however, is not overly problematic since a steady-state concentration is reached within a few minutes of continuous flow in most situations involving a reasonable range of flow rates and streamline volumes. It merely requires that sufficient time elapse to remove by flow ventilation any previously accumulated radon when the source was closed or operating at a different flow rate. Secondly, the approach of Eq. (9) assumes that the rate of removal of radon from the source by flow ventilation (or dilution) is rapid compared to its rate of generation. The rate constant *λ*_v_ for the rate of removal by ventilation in completely mixed, turbulent air is the quotient of the flow rate *f* by the internal volume *v* of the source, i.e., *λ*_v_ = *f*/*v*. Again, for any reasonable range of flow rates and internal source volumes *λ*_v_ ≫ *λ*_Rn_, and the conditional is easily satisfied. (It may be useful to note in passing, however, that for users of ^228^Th/^224^Ra sources to produce short-lived ^220^Rn (“thoron”) with decay constant *λ* = 0.0125 s^−1^, the rate constants *λ* and *λ*_v_ are often of comparable magnitude. In this case, all of the rate-of-change Bateman balance equations used to derive the steady-state solutions need to be modified by substituting (*λ* + *λ*_v_) for *λ*.)

The simple model of Eq. (9) implies that the steady-state ^222^Rn concentration in the streamline, *C*_Rn_, is just inversely proportional to the flow rate *f* through the source. The model, of course, is valid only if the emanation fraction *ϕ* is independent of flow rate, as well as of any other external variables. In addition, one can intuitively imagine that there must be only some reasonable operating range of flow rates for any realistic source. One could conceive that at exceedingly low flow rates, not all of the radon at the emanating surfaces of the source would be adequately entrained into the streamline for transport out of the source at constant time rates. Thus, because of possible variations in *ϕ* with flow rate or other external variables, as well as possible losses due to non-uniform transport at low flow rates, a calibration curve consisting of *C*_Rn_ as a function of flow rate *f* might very well exhibit deviations away from a strict 1/*f* proportionality.

The flow-through radium source used for the intercomparison was calibrated in terms of the available ^222^Rn concentration *C*_S_ maintained in a streamline at a constant flow rate *f*_4_. For these calibration measurements, the streamline was sampled by filling flow-through sample bulbs. The source was operated and the streamline sampled using the upper portion of the identical manifold described above in Sec. 2. This portion consisted of the source pump, appropriate control valves to adjust the flow rate through the source, a triad of valves to serve the inlet and outlet ports for the source and a bypass line, another triad of valves for the sampling ports, and flow meter F_4_.

The sample bulbs which were filled at known flow rates *f*_4_ were assayed for total ^222^Rn activity using the NIST primary ^222^Rn measurement system. This system is based on pulse-ionization-chamber (PIC) measurements calibrated against national ^226^Ra solution standards maintained by NIST, and has been described in detail previously by Collé et al. [2] and Hutchinson et al. [3]. The estimated uncertainty in the measurement of the total activity in the sample bulbs (corresponding to an approximate relative standard deviation) is approximately 0.7 % [3].

The volumes of the sample bulbs, used to obtain the activity concentration *C*_S_ from the total activity in the samples, were well known from replicate gravimetric determinations using high-purity water of known density at given temperatures. The sample bulbs were of various sizes ranging in volume from about 330 mL to 670 mL. The estimated overall relative standard uncertainty in the volumes is 0.2 % to 0.5 %, the magnitude of which was primarily controlled by the number of replicates.

Samples were collected and subsequently assayed for ^222^Rn to determine *C*_S_ over a range of flow rates from about 0.3 L · min^−1^ to 1.3 L · min^−1^. Continuous flow for a minimum of 10 min to 20 min (i.e., at least 10 complete air changes through the manifold) was maintained during sampling to flush the manifold and remove all previous air, and to insure adequate mixing. The precision of the flow-rate measurements during sampling was typically 0.02 % (relative standard deviation of the mean). Flow meter F_4_ was calibrated by its manufacturer (Matheson) and was stated to have a calibration accuracy of 1.0 % for air over the entire 0 L · min^−1^ to 3 L · min^−1^ flow rate range. Inasmuch as this uncertainty is presumed to correspond to some type of “maximum” confidence interval, the approximate relative standard deviation was taken to be 0.5 %.

**Fig. 3:**
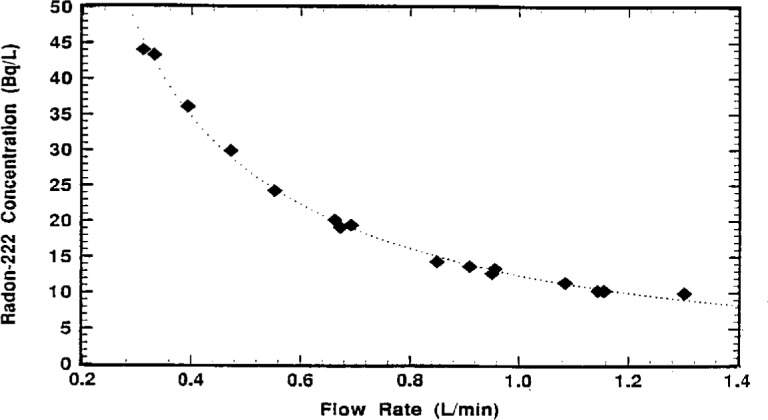
Calibration curve for the ^226^Ra flow-through source terms of the concentration of ^222^Rn in a streamline, *C*_S_, as a function of the flow rate in *f*_4_.

The calibration results for 16 independent determinations of *C*_S_ as a function of mean *f*_4_ are shown in Fig. 3. The data was fitted to obtain the *χ*^2^-minimized regression curve

$$C_{S}=a+\frac{b}{f_{4}}$$ (10)

with

$$a=-2.386(1\pm 16.0\%)\mathrm{Bq}\cdot L^{-1}$$

and

$$b=14.825(1\pm 1.5\%)\mathrm{Bq}\cdot L^{-1}$$

when *f*_4_ is expressed in units of L · min^−1^. The fitted residuals ranged from 0.1 % to 1.4 % with a correlation coefficient of *r* = 0.9968. Regressions using other fitting functions, including second- and higher-order polynomials in 1/*f*_4_ as well as exponentials, were inferior to that of Eq. (10). The uncertainties in *C*_S_ (for the flow rate range *f*_4_ = 0.3 L · min^−1^ to 1.3 L · min^−1^) are estimated to be 1.2 % to 2.6 % (relative standard uncertainty). This was obtained by propagating in quadrature the combined standard uncertainty in the sample assays for ^222^Rn concentration (0.7 % to 0.9 %), the flow meter standard uncertainty (0.5 %), and the standard uncertainty in *C*_S_ from the regression (0.77 % to 2.4 %). Refer to Table 7 in Sec. 6 for further detail.

The ^226^Ra source was a “Type RN-1025 Flow-Through Radon Gas Source” manufactured by Pylon Electronic Development Company (Ottawa, Canada). It was certified by Pylon in 1981 to contain 115.9 kBq ^226^Ra (decay corrected to October 1991) with a total calibration accuracy of ±4 % at the 99 % confidence level. The Pylon certificate also gave a continuously available ^222^Rn activity rate of 14.59 Bq · min^−1^; and indicated that in continuous flow the ^222^Rn activity concentration (in units of Bq · L^−1^) from the source could be calculated using the formula

$$C=\frac{14.59}{F}$$ (11)

over the flow rate (*F*) range of a few tenths of a L · min^−1^ up to 10 L · min^−1^. Comparison of Eqs. (10) and (11) indicate that there can be substantial differences between the calibration curve and that given by Pylon. Over even the small flow-rate range of 0.3 L · min^−1^ to 1.3 L · min^−1^, the Pylon valve exceeds that from the calibration curve by 3.4 % at *f*_4_ = 0.3 L · min^−1^, by 11 % at *f*_4_ = 0.7 L · min^−1^, by 17 % at *f*_4_ = 1.0 L · min^−1^, and by over 24 % at *f*_4_ = 1.3 L · min^−1^. These systematic differences have been consistently borne out by observations by NIST with this radium source over the past 8 years over even larger flow-rate intervals (up to 3.5 L · min^−1^).

It must be emphasized, however, that the calibration function of Eq. (10) is applicable for only this particular source and not meant to be applicable to any others. The model for the calibration function is phenomenalistically, not theoretically, based; it is meant to be practical for its intended use over the small flow-rate range. Nevertheless, the previous discussion suggested why there could be variations from the 1/*f* functionality. Even the functional form of Eq. (10) for this particular source is probably not adequate over a wider flow-rate range.

## 4. Radon-222 Standardized Additions

Fifteen sets of standardized additions using the methodology described in Sec. 2 were performed over a period of 5 d (October 9-13, 1991). The duration of each standard addition was 4 h, with the exception of #13 which was 3 h. The ^222^Rn activity concentrations, *C*_0_ of Eq. (6), for the standard additions ranged nominally from 2.5 Bq · m^−3^ to 35 Bq · m^−3^ (refer to Table 1). One of them (#4) had to be discarded from the analysis because a bypass valve on the manifold was inadvertently opened during the course of the addition which diverted the source flow stream from the main sampling line.

The standardized additions were analyzed in terms of calculating a mean *C*_0_ individually for each participating laboratory for each of their sampling/measurement intervals. For Lab E, the start and stop times of their 1 h sampling intervals were easily obtained from the abrupt changes in the flow rate *f*_1_ data (see Sec. 2). For Lab A, whose instrument recorded continuously and gave averaged results that were “smoothed” by a time constant of approximately 90 min [1], the entire 4 h (or 3 h in the case of #13) standard addition time interval was used to derive the mean *C*_0_. For Lab D, the mean *C*_0_ was calculated using the start and stop clock times for their 2 h sampling/measurement intervals which were submitted with their measurement results. As indicated earlier (Sec. 2), the flow-data clock time was cursorily intercompared and corrected to the standard (GMT) clock time used by Lab D.

To obtain a mean *C*_0_ for each standardized addition interval from some start time *t*_a_ to stop time *t*_z_ for each laboratories’ individual measurement cycle, the flow data could be analyzed in at least three different ways but all yield essentially the same numerical results.

The first approach consisted of calculating individual mean flow rates ${\bar{f}}_{4}$, ${\bar{f}}_{3}$, ${\bar{f}}_{5}$, and ${\bar{f}}_{1}$, over the given *t*_a_ to *t*_z_ interval. The mean ${\bar{f}}_{4}$ was then used to obtain a mean ${\bar{C}}_{S}$ from Eq. (10); and a mean dilution factor $\bar{D}$ was obtained from the individual flow-rate means using Eq. (7). The mean ${\bar{C}}_{0}$ was thereby approximated by the product of the derived ${\bar{C}}_{S}$ and $\bar{D}$ [Eq. (6)].

The second approach consisted of calculating the quantities *C*_S_(*t_i_*), *D*(*t_i_*), and *C*_0_(*t_i_*) from the flow-rate measurement data *f*_4_(*t_i_*), *f*_3_(*t_i_*), *f*_5_(*t_i_*), and *f*_1_(*t_i_*) at each *t_i_* measurement point (taken 10 s apart). The set of resulting *C*_0_(*t_i_*) values was then arithmetically averaged over the entire *t*_a_ to *t*_z_ interval to obtain the mean ${\bar{C}}_{0}$.

The third approach, considering the temporal variation in *C*_0_, consisted of numerically integrating the individual *C*_0_(*t_i_*) values at each point *t_i_* normalized by the total flow (from flow rate *f*_1_) in the main sampling line:

$${\bar{C}}_{0}=\frac{\int_{t_{a}}^{t_{z}}C_{0}(t)f_{1}(t)dt}{\int_{t_{a}}^{t_{z}}f_{1}(t)dt}=\frac{\sum_{t_{i}}C_{0}(t_{i})f_{1}(t_{i})}{\sum_{t_{i}}f_{1}(t_{i})}$$ (12)

Inspection of Eq. (12) reveals that the numerator represents the total ^222^Rn activity passing through the sampling line from time *t*_a_ to *t*_z_, while the denominator is the corresponding total volume.

The numerical differences in the mean *C*_0_ calculated by the three calculational approaches were in all cases negligible compared to the estimated relative standard deviations of the mean (*s_m_*) for *C*_0_ calculated by any one of the approaches. These differences, depending on the specific standardized addition in question, ranged from less than 0.01 % to about 2.0 % to 4.0 % in worst cases. The specific case-to-case variation in the precision in mean *C*_0_ may be seen in Tables 2, 3, and 4, which provide the calculated results of the standardized additions for the three participating laboratories. Estimates of *s_m_* for *C*_0_ may be obtained directly from *s_m_* for the mean dilution factor *D* since the latter is the singularly dominant source of variability in *C*_0_. These estimates of *s_m_* range from about 0.03 % to 3.0 % for Lab E, and from about 0.2 % to 3.6 % for Lab A and Lab D. The wide variations merely reflect the actual wide variations in the flow rates (mainly *f*_1_) from case to case as will be discussed shortly. Nevertheless, any differences due to the chosen calculational approach for a given case were clearly reflected and embodied within the estimated *s_m_* for that case.

The final calculational approach chosen for the intercomparison of standardized additions consisted of a combination of the first two approaches outlined above. It must be emphasized, however, that this choice was somewhat arbitrary since all of the approaches gave essentially identical results. None of the results and conclusions of the intercomparison would change as a result of a different calculational choice. For the adopted method chosen, the mean *C*_S_ was obtained from a mean *f*_4_ [Eq. (10)] as in the first approach; the mean dilution factor *D* [Eq. (7)] was derived as in the second approach; and the mean *C*_0_ was just the product of these values of *C*_S_ and *D*. These are the values tabulated in Tables 2, 3, and 4.

As indicated in the tables, the ^222^Rn activity concentrations *C*_0_ in the standardized sample additions ranged from approximately 2.5 Bq · m^−3^ to 35 Bq · m^−3^.

**Fig. 4:**
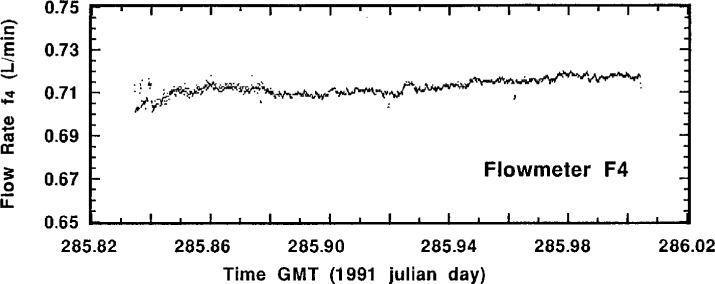
Typical flow rate *f*_4_ data as measured by meter F_4_ approximately every 10 s over an interval of 4 h for standard addition #11.

Perusal of the actual flow-rate measurement data for a typical standardized sample addition may be helpful in understanding the experimental aspects of the intercomparison and the quality of the results. Figures 4 through 7 give the flow-rate measurement data for *f*_4_, *f*_3_, *f*_5_, and *f*_1_, respectively, obtained during standardized addition #11. The illustrated data consists of nearly 1440 simultaneous measurements of the four flow rates, taken approximately every 10 s over an interval of 4 h.

**Fig. 5:**
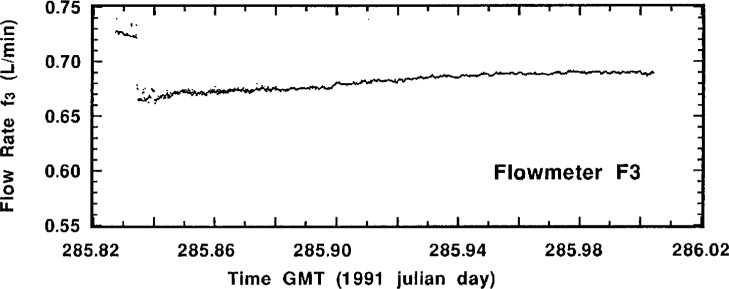
Typical flow rate *f*_3_ data as measured by meter F_3_ approximately every 10 s over an interval of 4 h for standard addition #11. The discontinuity in flow at the outset (from about 0.73 L · min^−1^ to 0.67 L · min^−1^) was an adjustment in the flow rate to change the dilution factor from standard addition #10 to #11 (see text).

**Fig. 6:**
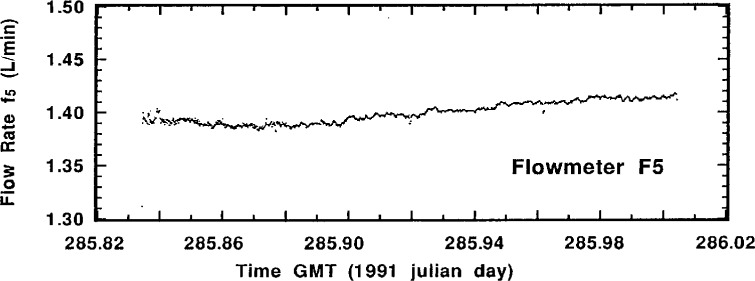
Typical flow rate *f*_5_ data as measured by meter F_5_ approximately every 10 s over an interval of 4 h for standard addition #11.

The means for the relatively stable flow rates *f*_4_, *f*_3_, and *f*_5_ were somewhat over determined and are very precise. Tables 2, 3, and 4 give the estimated relative standard deviations of the mean (*s_m_*) for *f*_4_, and these values of *s_m_* are typical of those for *f*_3_ and *f*_5_ as well. In fact, even these values are somewhat misleading for the inherent flow-rate variability in the middle of a standard addition. They are sometimes strongly influenced by abrupt changes in the flow rate that were made over the first 0.5 min to 2 min in adjusting the dilution factor in going from one standard addition to the next. Obviously, having a time delay between sequential additions would have obviated this shortcoming, but the rather costly nature of this intercomparison imposed time constraints. This kind of adjustment between standard additions is clearly shown in Fig. 5 where flow rate *f*_3_ was adjusted to vary the dilution factor from standard addition #10 to #11. Nevertheless, the data of Figs. 4 through 7 nicely illustrate the typical variations in flow over the course of a standard addition. The flow-rate data also often exhibited some slight, but gradual systematic drifts in flow rate over time. This effect is quite apparent in the data of *f*_3_ and *f*_5_ shown in Figs. 5 and 6. This is not believed to be a result of any electrical or signal “drift” in the flow meters themselves at constant flow, but rather is believed to have arisen from gradual changes in the settings of the flow control valves in the manifold due to mechanical vibrations at the site.

**Fig. 7:**
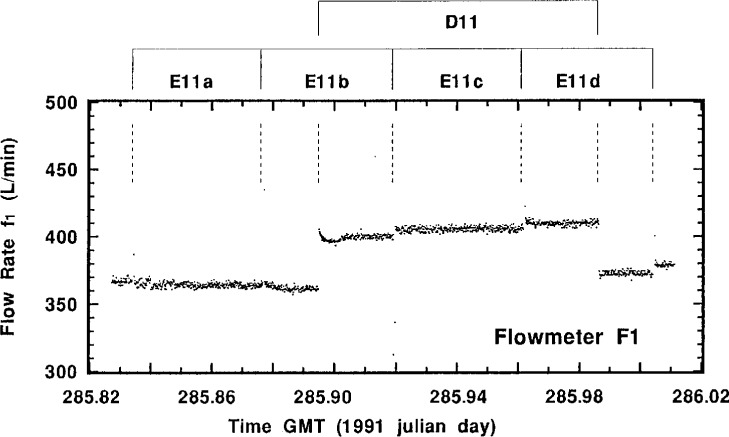
Typical flow rate *f*_1_ data as measured by meter F_1_ approximately every 10 s over an interval of 4 h for standard addition #11. The discontinuities are discussed in the text.

In contrast to the flow rates *f*_4_, *f*_3_, and *f*_5_, the data for flow rate *f*_1_, shown in Fig. 7, exhibit pronounced shifts. These marked changes arise as a result of the on and off cycling of the sampling pumps that were part of the measurement instruments for the three participating laboratories. The flow rate *f*_1_ at any time was the sum of the flow rates from the three laboratories’ pumps. Mean flow rates *f*_1_ calculated for each individual standardized addition over the course of all the additions ranged from approximately 365 L · min^−1^ to 450 L · min^−1^, with estimated relative standard deviations of the mean (*s_m_*) of essentially the same magnitude as that for the dilution factors *D* (see Tables 2, 3, and 4). The variability in *f*_1_ was the predominant contribution to the variability in the dilution factor *D*. The Lab E pump, which rapidly cycled on and off (over approximately 40 s) every hour was dominant, contributing about 365 L · min^−1^ to 395 L · min^−1^ of the total flow rate. The Lab D pump with a flow rate of about 40 L · min^−1^ to 45 L · min^−1^ sampled for 2 h, but at irregular intervals of about every 4 h to 5 h. The Lab A pump ran continuously with a flow rate close to 40 L · min^−1^ that increased to about 44 L · min^−1^ for 20 s every 10 min. The 4 intervals labelled E11a through E11d in Fig. 7 mark the pump sampling intervals for Lab E. The rapid drops in the flow rate as the Lab E pump shut down and then returned is very pronounced in the data, but is barely perceptible in Fig. 7 since each cycle only affects 3 or 4 out of the 1440 data values shown. The interval marked D11 in Fig. 7 represents the sampling interval for Lab D.

**Fig. 8:**
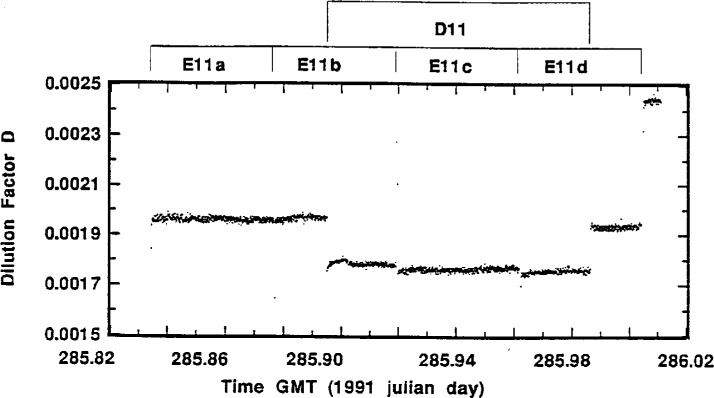
Dilution factor *D* as a function of time over the interval of 4 h for standard addition #11. The data values were calculated every 10 s using the flow rate data of Figs. 4 through 7.

The dilution factor *D* at each 10 s interval for the standard addition #11 flow data (Figs. 4 through 7) is given in Fig. 8. Of necessity, it exhibits the same marked discontinuities as the *f*_1_ data. It is apparent, for example, that the dilution factor *D* (and hence *C*_0_) is quite stable for the Lab E11a and E11c intervals; and is somewhat less so for the Lab D11 interval because of the two disruptions by the Lab E pump. The intervals E11b and E11d exhibit pronounced step functions in the dilution factor as the Lab D pump went on and off. These evident and very different kinds of variations in the dilution factor are also manifested by the magnitude of their respective estimated relative standard deviations of the mean (*s_m_*).

Consider the respective cases for the three laboratories.

Table 2 indicates that for the stable E11a and E11c intervals, *s_m_* for dilution factor *D* is 0.02 % and 0.06 %; while for intervals E11b and E11d, with the step function in *D*, *s_m_* is 0.26 % and 0.25 %, respectively. Intuitively, one might expect *s_m_* for mean *D* for these cases with a drastic step function in *D* to be even larger than that given. This estimated *s_m_*, however, is easily derived and verified from its component parts. For a step function with mean *m*_1_ and *s_m_*_1_ at the low plateau having a time duration Δ*t*_1_, and with mean *m*_2_ and *s_m_*_2_ at the high plateau for time duration Δ*t*_2_, the mean *m* over the entire (Δ*t*_1_ + Δ*t*_2_) duration is approximately

$$m≃\frac{\Delta t_{1}(m_{1})+\Delta t_{2}(m_{2})}{\left(\Delta t_{1}+\Delta t_{2}\right)};$$ (13)

and the relative standard deviation of the mean for *m*, obtained by appropriately adding the variances of *m*_1_ and *m*_2_, is

$$\begin{array}{l} s_{m}=\\ \sqrt{{\left[\frac{\Delta t_{1}}{\Delta t_{1}+\Delta t_{2}}\right]}^{2}{\left(\frac{m_{1}}{m}\right)}^{2}s_{m_{1}}^{2}+{\left[\frac{\Delta t_{2}}{\Delta t_{1}+\Delta t_{2}}\right]}^{2}{\left(\frac{m_{2}}{m}\right)}^{2}s_{m_{2}}^{2}}. \end{array}$$ (14)

Clearly, the mean *m* is proportionately-distant between the low and high plateaus of the step function, and the negative residuals at the low plateau are counterbalanced by all of the positive residuals at the high plateau. Furthermore, the step function in dilution factor *D* is inherently bimodally distributed (i.e., a bivariate distribution) in these cases, and *s*_m_ for the overall mean *m* is an indescriptive and non-robust statistical estimator of the variability in *D*. The estimated *s_m_*, nevertheless, if anything, overestimates the true dispersion in the two bimodal means. In view of the invariance (within statistical variations) of the three different calculational approaches to obtain a mean *C*_0_ (Sec. 4), it is clear that even with the step function in *D* (and thereby in *C*_0_), the adopted treatment is adequate.

The mean dilution factor *D* for interval D11 has a *s_m_* for *D* of 1.1 % (Table 4). Although it is not as apparent in the illustrated data of Fig. 8, its magnitude is strongly influenced by the two abrupt flow discontinuities when the Lab E pump went off and on.

Over the entire 4 h interval of Fig. 8, as applicable for calculation of the Lab A dilution factor mean *D*, *s_m_* for *D* was 0.9 % (Table 3).

## 5. Confirmatory Measurements

The dimensionless dilution factors *D* [Eq. (7)], used to obtain the values of *C*_0_ for the intercomparison, consist of flow rate ratios, and therefore are more dependent on the relative flow meter responses rather than on individual flow meter calibrations *per se*. Although the matched flow meters F_4_, F_3_, and F_5_ in the NIST manifold were previously intercompared and also presumably based on similar and relatable calibrations by the manufacturer (Matheson), the design of the manifold (Fig. 1) and the simultaneous flow-rate measurements allowed a direct, *in situ* intercomparison of the flow meters at the test site.

Inspection of Figs. 1 and 2 indicates that when *f_x_* = 0, then, in principle, (*f*_4_ + *f*_3_) = *f*_5_. This provided the opportunity to intercompare the flow-meter responses for the following conditions

$$\begin{array}{l} \frac{f_{4}}{f_{5}}=1\;\text{with}\;f_{3}=0\\ \frac{f_{3}}{f_{5}}=1\;\text{with}\;f_{4}=0\\ \frac{f_{4}+f_{3}}{f_{5}}=1. \end{array}$$ (15)

All three conditions were tested in Bermuda during the course of the intercomparison. The results are given in Table 5. Mean values for the flow rates were obtained from the 10 s measurement data averaged over periods of from 20 min to nearly 60 min. All of the estimated relative standard deviations of the mean for the flow rates were in the range 0.01 % to 0.04 %. As indicated, all of the flow-ratio intercomparisons are within ± 1 %, which is well within the range of the expected statistical variations for meters with an assumed relative standard uncertainty of 0.5 % (see Sec. 3 discussion).

Regrettably, it was not possible to independently verify by intercomparison the relative response of flow meter F_1_ to the others. The repercussions of this shortcoming as it affects the results and conclusions of the intercomparison of standardized additions will be addressed in Part II of this series [1].

The conformity of the flow rates at sequential locations in the streamlines of the manifold also demonstrated the absence of any serious leaks in the manifold’s construction.

Two types of *in situ* confirmatory tests of the operation of the NIST manifold to deliver known ^222^Rn activity concentrations at the site in Bermuda were performed. Both involved collecting “grab samples” from the manifold using glass sampling bulbs identical to those used to perform the ^226^Ra source calibration. The samples were returned to NIST for assay, again using a PIC measurement procedure identical to that used in calibrating the radium source (see Sec. 3). The assayed ^222^Rn activity concentrations in the samples were decay corrected to the time of sample collection, and this corrected concentration *C*_T_ was compared to that concentration *C*_P_ predicted from the operation of the manifold.

The first kind of test involved samples collected at the sampling port directly downstream from the radium source (see Fig. 1). The activity concentrations in these samples are expected to be that given by the source calibration [*C*_S_ of Eq. (10)] as a function of the flow rate *f*_4_. Three such samples were collected during standard additions #7, #10, and #14, with the results summarized in Table 6. An additional two samples were collected, but were unfortunately destroyed in transit to NIST.

The second kind of test was performed at the conclusion of the standardized additions when the NIST manifold was disconnected from the main sampling line. Two samples were collected directly at the outlet of the manifold (see Fig. 1) after meter F_5_ and just as it would have entered the main line before meter F_1_. This confirmatory test was made to demonstrate the efficacy of mixing the *f*_4_ and *f*_3_ flows, and the (*f*_4_ + *f*_3_) dilution calculation. In this case, the predicted concentration *C*_P_ is given by Eq. (3), where *C*_S_ is diluted by the factor *f*_4_/(*f*_4_ + *f*_3_). The results for theses two samples, labelled ND and NF, are also presented in Table 6.

The mean flow rates for *f*_4_ and *f*_3_ used to obtain the predicted concentration *C*_S_ and diluting factor *f*_4_/(*f*_4_ + *f*_3_) were obtained from the 10 s flow-rate data averaged over intervals of approximately 10 min that immediately preceded the closing of the sample bulbs. The estimated relative standard deviation of the mean for *f*_4_ and *f*_3_ was, in all cases, less than 0.01 %. The mean values averaged over shorter intervals (down to 3 min) and longer intervals (up to 20 min) were invariant.

Comparison of the assayed concentrations *C*_T_ and predicted values *C*_P_ in Table 6 indicates an overall agreement to slightly over 1 %, with no significant differences between the two kinds of samples. The mean value of *C*_P_/*C*_T_ = 0.987 has an estimated relative standard deviation of the mean (*s*_m_) of 0.49 %. This uncertainty is roughly the same magnitude as that expected for the derived uncertainty in *C*_S_ as given earlier (Sec. 3). As indicated, a very slight negative bias in *C*_P_/*C*_T_ may be suggested, particularly for the three direct *C*_S_ samples. Yet, even if this bias exists, the statistical variations and small number of samples does not allow its confirmation. It should be noted that the *C*_P_/*C*_T_ comparison given in Table 6 neglected any contribution of radon activity from ambient air. In actuality, a comparison of (*C*_P_ + *C*_A_)/*C*_T_ (where *C*_A_ is the ambient ^222^Rn activity concentration at the time of sample collection) would be more valid. However, *C*_A_ is believed to be ⩽ 0.003 Bq · L^−1^ for any of the samples, and should therefore have a negligible effect (⩽ 0.03 % of *C*_T_) on the results. This limit is based on the maximum ambient concentration that was measured by any of the participating laboratories at any time during the intercomparison (see Ref. [1]).

## 6. Uncertainty Analysis

A complete analysis of the measurement uncertainties for the ^226^Ra source calibration and ^222^Rn activity concentrations in the standardized sample additions is outlined *in extenso* in Table 7.

The uncertainty analysis procedure follows the normal conventions of the NIST Radioactivity Group which for the most part are compatible with those adapted by the principal international metrology standardization bodies [4,5]. All individual uncertainty components are expressed in terms of estimated (experimental) standard deviations (or standard deviations of the mean where appropriate) or quantities assumed to correspond to standard deviations, irrespective of the method used to evaluate their magnitude. A propagated or “combined standard uncertainty” is expressed as an estimated standard deviation which is equal to the positive square root of the total variance obtained by summing all variance and covariance components, however evaluated, using the law of propagation of uncertainty for the specific mathematical function given by the model of the measurement procedure. By convention in this laboratory (at the time of this intercomparison),^2^ the combined standard uncertainty is expanded by a “coverage factor” of 3 to obtain an “expanded uncertainty” (or “overall uncertainty” [*sic*]) which is assumed to provide an uncertainty interval having a high level of confidence of roughly 95 % to 99 %.

The analysis of the propagated uncertainty in the concentration *C*_S_ exiting the radium source as a function of the flow rate *f*_4_ was discussed previously in Sec. 3.

To derive the uncertainty in the concentration *C*_0_ for the standardized sample additions from the uncertainty in *C*_S_, one must consider the additional uncertainty components due to: (1) the mean for flow rate *f*_4_ for the given addition; (2) the mean dilution factor *D*; (3) the flow-meter calibrations; and (4) the timing of the addition which determines the start and stop points of the numerical calculation of the *f*_4_ and *D* means.

The statistical estimators (*s_m_*) for *f*_4_ and *D* were treated and discussed at considerable length in the example for addition #11 in Sec. 4. The values listed in Table 7 have wide ranges exceeding an order of magnitude. The reasons for the wide variations were also addressed in Sec. 4.

The uncertainties in the flow-meter calibrations, again expressed as relative standard deviations, were obtained by taking one half of the manufacturers’ stated calibration accuracies since the latter are presumed to correspond to a high level of confidence. The uncertainty in the timing of the standardized addition is also highly variable depending on the specific case. The uncertainties (*Δ*) in the actual start (*t*_a_) and stop (*t*_z_) times *per se* are not critical, but rather it is the uncertainty in calculating the means *f*_4_ and *D* over the interval from $\left(t_{a}\pm \Delta_{t_{a}}\right)$ to $\left(t_{z}\pm \Delta_{t_{z}}\right)$ that is of importance. The additions for Lab D were largely free of this uncertainty. Their measurement intervals were (with one exception) completely overlapped by the 4 h or 3 h standard addition intervals. Shifting *t*_a_ and *t*_z_ by even several minutes in either direction has almost negligible effects (< 0.1 %) on the results of the numerical integrations for the Lab D additions. The Lab A additions utilized the calculational results for the entire 4 h or 3 h sample intervals. It is conceivable that the clock times for their sampling intervals of 1 h differed from the assumed *t*_a_ and *t*_z_ times by 1 min to 2 min. This presented the real possibility of having overflows of radon from one presumed standardized addition interval to another in the case of adjacent addition intervals. Perhaps, this kind of source of inaccuracy should be considered to fall within the category of a “blunder” rather than an evaluatable uncertainty component. Yet, considering the quality and nature of their “smoothed” measurement results and the somewhat equivocable, attendant analysis of their data that was thereby required [1], this uncertainty component was truly negligible in terms of any interpretations of the measurement results and conclusions of the intercomparison. The abrupt flow interruptions by the pump for Lab E served as an internal timer to determine the start and stop times of the additions (see Secs. 2 and 4) so, in principle, their additions were nearly perfectly synchronized in terms of real clock times. However, even the small 15 s to 20 s $\Delta_{t_{a}}$ and $\Delta_{t_{z}}$ intervals, which had accompanying large fluctuations in the flow rates just at the *t*_a_ and *t*_z_ times, resulted in the largest uncertainties in *f*_4_ and *D*. These approached 0.3 % of *f*_4_ in worst cases.

The above component uncertainties may be propagated, as done in Table 7, to form the uncertainty in *C*_0_. It ranges from about 2.0 % to 4.4 % of *C*_0_, or about 6 % to 13 % at the “overall uncertainty” level. This is the uncertainty associated with the ^222^Rn activity concentration in the standardized sample additions, exclusive of any contributions from ^222^Rn in the ambient air. Considering the uncertainty associated with the uncertainty $\delta_{C_{0}}$ itself (as well as the largely unknown degrees of freedom and the unknown probability distributions for the component quantities), individual values of $\delta_{C_{0}}$ were not propagated for each of the 83 standardized sample additions listed in Tables 2, 3, and 4. That kind of arithmetic exercise was deemed to have little significant value. The detailed uncertainty analysis presented here resulted in a range of 6 % to 13 % for the “overall uncertainty” in *C*_0_, which justifiably may be considered to be in the general range of approximately 10 %.

Now, to treat the uncertainty in the ^222^Rn activity concentration *C*_1_ in the main sampling line as sampled by the participating laboratories, one must consider the contribution due to the ambient concentration, *C*_A_. Recalling that *C*_1_ = *C*_0_ + *C*_A_ [Eq. (5)], the uncertainty in *C*_1_ is given by the sum of the variances $u_{C_{1}}^{2}=u_{C_{0}}^{2}+u_{C_{A}}^{2}$, which yields

$$\delta_{C_{1}}=\sqrt{{\left(\frac{C_{0}}{C_{1}}\right)}^{2}\delta_{C_{0}}^{2}+{\left(\frac{C_{A}}{C_{1}}\right)}^{2}\delta_{C_{A}}^{2}}$$ (16)

when the uncertainties (*δ*) are expressed as relative standard deviations. Obviously, the uncertainty in $\delta_{C_{1}}$ can vary extensively depending on the magnitude of the concentration ratio *C*_0_/*C*_A_ and the uncertainty in the ambient concentration $\left(\delta_{C_{A}}\right)$. Table 7 illustrates this for the hypothetical case of an assumed ambient concentration uncertainty $\left(\delta_{C_{A}}\right)$ of 50 % at three *C*_0_/*C*_A_ ratios. For *C*_0_/*C*_A_ = 10, the uncertainty $\delta_{C_{1}}$ increases by about a factor of 1.5 to 2.5 times $\delta_{C_{0}}$; while for *C*_0_/*C*_A_ = 100, there is negligible difference between $\delta_{C_{1}}$ and $\delta_{C_{0}}$. Throughout the course of the intercomparison for all standardized sample additions, the ratio *C*_0_/*C*_A_ was estimated to lie in the range 10 < *C*_0_/*C*_A_ < 1000 [1].

## 7. Conclusions

This work provided a standardized reference basis for the first international intercomparison of ^222^Rn detectors used in marine-atmospheric studies. The work went beyond serving the needs of this particular intercomparison. More importantly, it also demonstrated the broader utility of the calibration protocol and the methodology for the standardized sample additions that were developed for it.

The intercomparison not only provided a basis for comparing the measurement results and performance of different instruments of various participating laboratories, but more so, provided a common reference to these laboratories and provided the possibility for an *in situ* intercalibration that could be related to U.S. national ^226^Ra standards. Most environmental measurement intercomparisons of field instruments in actual use merely rely on evaluating the relative performance of the participants, or some comparison to the pooled results. This exercise demonstrated, for the very first time, the capability of providing a standardized reference basis even for such low-level, field-measurement intercomparisons.

The standardized sample additions of known, but undisclosed (“blind”) ^222^Rn activity concentration used for this intercomparison ranged from approximately 2.5 Bq · m^−3^ to 35 Bq · m^−3^, and had “overall uncertainties” (that can be related to national standards) in the general range of approximately 10 % at an assumed three standard deviation uncertainty interval. As will be presented and discussed in the second paper of this series [1], the ^222^Rn activity concentrations in Bermudian ambient air over the course of the intercomparison are understood to range from < 0.01 Bq · m^−3^ to roughly 2 Bq · m^−3^. Thus, there was nearly a complete and compatible overlap between the concentrations in the standardized sample additions and in ambient air.

The developed methodologies presented here could, of course, be adopted with slight modifications to cover other ^222^Rn concentration ranges and other applications, and could be employed in many other types of ^222^Rn environmental, field-measurement intercomparisons.

## Figures and Tables

**Table 1:** NIST standardized sample additions

No.	Approximate time		Nominal range for the ^222^Rn activity concentration
	Date—1991	Hours (GMT)	(Bq · m^−3^)
1	9 October	1900 – 2300	33 to 37
2	10 October	0900 – 1300	28 to 30
3	10 October	1400 – 1800	29 to 31
4	11 October	0900 – 1300	discarded
5	11 October	1400 – 1800	5.3 to 6.2
6	11 October	1800 – 2200	20 to 23
7	11–12 October	2200 – 0200	3.9 to 5.0
8	12 October	0200 – 0600	3.8 to 4.1
9	12 October	1200 – 1600	7.2 to 7.9
10	12 October	1600 – 2000	12 to 14
11	12 October	2000 – 0000	32 to 36
12	13 October	0000 – 0400	30 to 33
13	13 October	1200 – 1500	15 to 16
14	13 October	1500 – 1900	5.4 to 5.8
15	13 October	1900 – 2300	2.4 to 2.6

**Table 2:** NIST standard additions for Lab E

	Flow rate *f*_4_		^222^Rn Conc. *C*_S_	Dilution factor *D*		^222^Rn Conc. *C*_0_^b^
No.	Mean (L · min^−1^)	*s_m_*^a^ (%)	(Bq · L^−1^)	Mean (× 10^−3^)	*s_m_*^a^ (%)	(Bq · m^−3^)
1	0.335	0.02	41.92	0.854	0.19	35.81
	0.333	0.02	42.15	0.796	0.03	33.55
	0.332	0.01	42.25	0.778	0.02	32.87
	0.328	0.01	42.83	0.865	0.04	37.07
2	1.200	0.01	9.968	2.919	0.18	29.09
	1.201	0.03	9.960	2.804	0.04	27.92
	1.194	0.01	10.03	2.874	0.14	27.82
	1.186	0.01	10.11	3.014	0.03	30.48
3	1.173	0.02	10.25	2.909	0.22	29.81
	1.175	0.02	10.24	2.792	0.04	28.59
	1.166	0.02	10.33	2.946	0.25	30.44
	1.175	0.11	10.23	3.052	0.31	31.23
5	0.985	0.04	12.67	0.422	0.04	5.349
	0.981	0.01	12.72	0.430	0.05	5.467
	0.979	0.01	12.75	0.454	0.05	5.787
	0.990	0.03	12.58	0.460	0.08	5.782
6	0.768	0.06	16.91	1.298	0.41	21.94
	0.770	0.02	16.87	1.211	0.04	20.43
	0.773	0.02	16.79	1.294	0.27	21.74
	0.772	0.03	16.82	1.300	0.04	21.87
7	0.645	0.41	20.60	0.243	3.0	5.013
	0.664	0.02	19.96	0.207	1.1	4.141
	0.668	0.01	19.82	0.199	0.06	3.946
	0.670	0.01	19.73	0.210	0.10	4.143
8	0.676	0.01	19.54	0.208	0.04	4.064
	0.669	0.02	19.78	0.201	0.14	3.978
	0.671	0.01	19.71	0.195	0.04	3.833
	0.671	0.01	19.70	0.198	0.15	3.898
9	1.051	0.12	11.72	0.627	0.63	7.350
	1.042	0.02	11.84	0.607	0.03	7.181
	1.019	0.05	12.16	0.619	0.19	7.524
	1.026	0.02	12.07	0.654	0.07	7.885
10	0.958	0.03	13.08	1.025	0.15	13.41
	0.944	0.01	13.32	0.930	0.03	12.39
	0.944	0.01	13.31	0.952	0.05	12.67
	0.953	0.02	13.17	1.041	0.11	13.70
11	0.710	0.02	18.49	1.961	0.02	36.25
	0.710	0.01	18.50	1.863	0.26	34.46
	0.713	0.02	18.40	1.764	0.06	32.47
	0.717	0.01	18.29	1.831	0.25	33.49
12	0.905	0.01	14.00	2.391	0.02	33.48
	0.909	0.01	13.92	2.278	0.12	31.71
	0.916	0.02	13.80	2.051	0.03	28.30
	0.907	0.01	13.95	2.124	0.03	29.64
13	0.916	0.10	13.80	1.072	0.16	14.79
	0.920	0.02	13.73	1.142	0.14	15.68
	0.922	0.01	13.69	1.144	0.02	15.66
14	1.073	0.15	11.42	0.505	1.33	5.770
	1.078	0.01	11.37	0.477	0.03	5.422
	1.079	0.01	11.36	0.499	0.21	5.671
	1.081	0.01	11.33	0.513	0.03	5.811
15	1.146	0.03	10.56	0.242	0.58	2.556
	1.145	0.01	10.56	0.225	0.06	2.371
	1.143	0.01	10.59	0.225	0.85	2.383
	1.138	0.01	10.65	0.232	0.10	2.465

a Relative standard deviation of the mean.

b The relative “overall uncertainties” ranged from 6.0 % to 13.0 % (see Table 7).

**Table 3:** NIST standard additions for Lab A

	Flow rate *f*_4_		^222^Rn Conc. *C*_S_	Dilution factor *D*		^222^Rn Conc. *C*_0_^b^
No.	Mean (L · min^−1^)	*s_m_*^a^ (%)	(Bq · L^−1^)	Mean (× 10^−3^)	*s_m_*^a^ (%)	(Bq · m^−3^)
1	0.332	0.02	42.30	0.825	0.21	34.91
2	1.195	0.02	10.02	2.915	0.20	29.20
3	1.172	0.03	10.26	2.935	0.21	30.11
5	0.984	0.02	12.68	0.480	1.9	6.09
6	0.771	0.02	16.84	1.338	2.0	22.53
7	0.662	0.11	20.02	0.217	1.0	4.35
8	0.672	0.01	19.68	0.206	1.2	4.06
9	1.035	0.05	11.94	0.643	0.89	7.68
10	0.950	0.02	13.22	1.003	0.57	13.27
11	0.713	0.01	18.42	1.900	0.88	35.01
12	0.909	0.01	13.92	2.232	0.33	31.06
13	0.919	0.03	13.74	1.149	1.1	15.78
14	1.078	0.04	11.37	0.510	0.86	5.80
15	1.143	0.01	10.59	0.238	1.9	2.52

a Relative standard deviation of the mean.

b The relative “overall uncertainties” ranged from 6.0 % to 13.0 % (see Table 7).

**Table 4:** NIST standard additions for Lab D

	Flow rate *f*_4_		^222^Rn Conc. *C*_S_	Dilution factor *D*		^222^Rn Conc. *C*_0_^b^
No.	Mean (L · min^−1^)	*s_m_*^a^ (%)	(Bq · L^−1^)	Mean (× 10^−3^)	*s_m_*^a^ (%)	(Bq · m^−3^)
1	0.332	0.02	42.21	0.792	0.27	33.43
2	1.198	0.02	9.99	2.844	0.26	28.41
3	1.174	0.02	10.25	2.823	0.26	28.93
5	0.983	0.02	12.70	0.490	3.2	6.22
6	0.771	0.02	16.85	1.291	3.0	21.76
7	0.666	0.01	19.88	0.205	0.81	4.08
8	0.671	0.01	19.72	0.202	2.3	3.98
9	1.040	0.04	11.88	0.619	0.94	7.35
10	0.944	0.01	13.32	0.964	0.94	12.84
11	0.714	0.02	18.39	1.814	1.09	33.36
12	0.912	0.02	13.88	2.104	0.31	29.20
13	0.917	0.09	13.78	(0.612)^c^	1.2	(8.43)^c^
14	1.079	0.01	11.36	0.488	0.91	5.54
15	1.143	0.01	10.58	0.234	3.6	2.48

a Relative standard deviation of the mean.

b The relative “overall uncertainties” ranged from 6.0 % to 13.0 % (see Table 7).

c This standard addition (#13) for Lab D was normalized by the ratio 0.5583 to account for a partial standard addition during the measurement interval. The standard addition was made for only the last 67 min of the 120 min measurement interval.

**Table 5:** *In situ* intercomparison of the flow meters F_4_, F_3_, and F_5_

	Flow rates (L · min^−1^)						
Test conditions	*f*_3_ Mean	*s_m_*^a^ (%)	*f*_4_ Mean	*s_m_*^a^ (%)	*f*_5_ (Mean)	*s_m_*^a^ (%)	Flow ratio
*f*_4_ = 0	0.770	0.02			0.766	0.03	$\frac{f_{3}}{f_{5}}=1.005$
*f*_3_ ≃ *f*_5_							
*f*_3_ = 0			0.793	0.01	0.797	0.03	$\frac{f_{4}}{f_{5}}=0.995$
*f*_4_ ≃ *f*_5_			0.919	0.01	0.927	0.01	= 0.991
			1.078	0.04	1.076	0.02	= 1.002
*f*_3_ + *f*_4_ ≃ *f*_5_	0.681	0.03	0.713	0.01	1.400	0.02	$\frac{f_{3}+f_{4}}{f_{5}}=0.996$

a Estimated relative standard deviation of the mean.

**Table 6:** Results of the confirmatory measurements for ^222^Rn activity concentration collected *in situ* at Bermuda and assayed at NIST

Sample	*f*_4_ (L · min^−1^)	*C*_S_^a^ (Bq · L^−1^)	*f*_3_ (L · min^−1^)	$\left[\frac{f_{4}}{f_{4}+f_{3}}\right]$	*C*_P_^b^ (Bq · L^−1^)	*C*_T_^c^ (Bq · L^−1^)	*C*_P_/*C*_T_
N10	0.9572	13.10	(0)	(1)	13.10	13.27	0.9873
N14	1.0840	11.29	(0)	(1)	11.29	11.43	0.9878
N7(b)	0.6924	19.03	(0)	(1)	19.03	19.43	0.9792
ND	0.4715	29.06	0.6948	0.4043	11.75	11.69	1.0049
NF	0.3932	35.32	0.6983	0.3602	12.72	13.01	0.9779
					Number *C*_P_/*C*_T_ mean *s_m_* (%)^d^		5
							0.9874
							0.49

a See Eq. (10).

b *C*_P_ is the predicted ^222^Rn activity concentration given by *C*_P_ = [*f*_4_/(*f*_4_ + *f*_3_)]*C*_S_.

c *C*_T_ is the assayed, decay-corrected ^222^Rn activity concentration at the “grab sample” time of collection, corresponding to that expected for *C*_P_; refer to text for details.

d Relative standard deviation of mean in percent.

**Table 7:** Analysis of uncertainties for the ^222^Ra source calibration and ^222^Rn concentrations in the standardized additions

Source of uncertainty		Component uncertainty^a^	Propagated uncertainty^a^	Typical “overall uncertainty”^b^
Precision of radon measurement in sample bulbs by PIC (*δ_i_*)		0.05 to 0.2	—	—
PIC calibration (*δ_i_*)		0.7^c^	—	
Volume sample bulbs (*δ_i_*)		0.2 to 0.5	—	—
Radium and radon decay corrections (*δ_i_*)		< 0.01	—	—
Radon concentration in sample $\left(\delta_{S}=\sqrt{\sum_{i}\delta_{i}^{2}}\right)$		—	0.73 to 0.88	2.2 to 2.6
Precision flow rate during sampling (*δ_j_*)		0.02	—	—
Flow meter calibration (*δ_j_*)		0.5^d^	—	
Flow rate $\left(\delta_{F}=\sqrt{\sum_{j}\delta_{j}^{2}}\right)$		—	0.5	—
Fit (regression) of radon concentration as a function of flow rate (*δ*_R_)		0.77 to 2.4	—	—
Radon concentration (*C*_S_) from source at given flow rate $\left(\delta_{C_{S}}=\sqrt{\delta_{S}^{2}+\delta_{F}^{2}+\delta_{R}^{2}}\right)$		—	1.2 to 2.6	3.5 to 7.8
Timing errors for standardized addition (*δ_k_*)		0.1 to 0.3	—	—
Overall precision in diulution factor from flow rate measurement (*δ_k_*)		0.2 to 3.0	—	—
Calibration flow meters F_3_, F_4_, and F_5_ (*δ_k_*)		0.5^d^	—	
Calibration flow meter F_1_ (*δ_k_*)		1.4^e^	—	
Dilution factor $\left(\delta_{D}=\sqrt{\sum_{k}\delta_{k}^{2}}\right)$		—	1.6 to 3.5	—
Precision flow rate *f*_4_ for std. addition $\left(\delta_{f_{4}}\right)$		0.1 to 0.4	—	—
Radon concentration (*C*_0_) in std. addition (excluding ambient conc.) $\delta_{C_{0}}=\sqrt{\delta_{C_{S}}^{2}+\delta_{D}^{2}+\delta_{f_{4}}^{2}}$		—	2.0 to 4.4	6.0 to 13
Radon concentration (*C*_A_) for ambient air $\left(\delta_{C_{A}}\right)$		Assumed 50	—	—
Radon concentration (*C*_1_) for std. addition (including ambient conc.) $\delta_{C_{1}}=\sqrt{{\left(\frac{C_{A}}{C_{1}}\right)}^{2}\delta_{C_{A}}^{2}+\left(\frac{C_{0}}{C_{1}}\right)\delta_{C_{0}}^{2}}$				
*Variable:*	For *C*_0_/*C*_A_ = 100	—	2.0 to 4.4	6.1 to 13
	For *C*_0_/*C*_A_ = 25	—	2.7 to 4.6	8.2 to 14
	For *C*_0_/*C*_A_ = 10	—	4.9 to 6.1	15 to 18

a The estimated component and propagated uncertainties are assumed to correspond to an approximate relative standard deviation (or standard deviation of the mean) expressed in percent.

b The “overall uncertainty” (or expanded uncertainty with coverage factor *k* = 3) is taken to be three times the propagated uncertainty in percent.

c See Refs. [2] and [3].

d Corresponds to one half the vendor’s (Matheson) stated calibration accuracy.

e Corresponds to one half the vendor’s (TSI, Inc.) stated calibration “tolerance.”

## Footnotes

1 Certain commercial equipment, instruments or materials are identified in this paper to foster understanding. Such identification does not imply recommendation or endorsement by the National Institute of Standards and Technology, nor does it imply that the materials or equipment identified are necessarily the best available for the purpose.

2 NIST has since uniformly adopted a policy of using a coverage factor of *k* = 2.
